# Internal Dynamics of Ionic Liquids over a Broad Temperature Range—The Role of the Cation Structure

**DOI:** 10.3390/ma15010216

**Published:** 2021-12-28

**Authors:** Danuta Kruk, Mariusz Jancelewicz, Adam Klimaszyk, Roksana Markiewicz, Zbigniew Fojud, Stefan Jurga

**Affiliations:** 1 Department of Physics and Biophysics, University of Warmia & Mazury in Olsztyn, Oczapowskiego 4, 10-719 Olsztyn, Poland; 2 NanoBioMedical Centre, Adam Mickiewicz University, Wszechnicy Piastowskiej 3, 61-614 Poznan, Poland; mariusz.jancelewicz@amu.edu.pl (M.J.); adam.klimaszyk@amu.edu.pl (A.K.); roksana.markiewicz@amu.edu.pl (R.M.); stjurga@amu.edu.pl (S.J.); 3 Department of MacromoLecular Physics, Faculty of Physics, Adam Mickiewicz University, Uniwersytetu Poznanskiego 2, 61-614 Poznan, Poland; zbigniew.fojud@amu.edu.pl

**Keywords:** ionic liquids, dynamics, nuclear magnetic resonance, relaxation

## Abstract

^1^H and ^19^F spin-lattice relaxation experiments have been performed for a series of ionic liquids sharing the same anion: bis(trifluoromethanesulfonyl)imide but including cations of different alkyl chain lengths: butyltriethylammonium, triethyloctylammonium, dodecyltriethylammo-nium and hexadecyltriethylammonium. The studies have been carried out in the temperature range from 383 to 108 K at the resonance frequency of 200 MHz (for ^1^H). A quantitative analysis of the relaxation data has revealed two dynamical processes for both kinds of ions. The dynamics have been successfully modeled in terms of the Arrhenius law. The timescales of the dynamical processes and their temperature evolution have been discussed in detail, depending on the structure of the cation.

## 1. Introduction

Ionic liquids are composed with organic cations and inorganic or organic anions. The compounds exhibit attractive physical and chemical properties, such as low volatility, flammability, high thermal and chemical stability. Properties of ionic liquids can be tailored by varying cation and anion structure, which in turn causes changes to the structure and dynamics of these compounds as a whole [1,2,3]. This makes them a unique class of systems of various applications: as electrolytes [2,4], solvents, and catalysts for chemical synthesis [5] or bioactive agents and pharmaceuticals [6,7] among others. In the context of both the fundamental and applied sciences, it is the dynamics of ionic liquids that raise a lot of interest. As far as the fundamental aspects are concerned, one is interested in the influence of inter-ionic electrostatic interactions on the mechanism of the ionic motion, the effect of even small changes in the ionic structure on the dynamical processes, or changes in the ionic motion upon phase transitions. The dynamical properties of the ions determine their application performance by determining, for instance, the conductivity of ionic liquids.

Discussing the motion of ions constituting ionic liquids, one thinks about their translation diffusion, rotational dynamics, and internal motion. Nuclear Magnetic Resonance (NMR) methods are often exploited to enquire into the dynamical properties of ionic liquids. The direct NMR method of measuring translation diffusion coefficients exploits magnetic field gradients [8,9] and is referred to as NMR diffusometry. The underlying principle of NMR diffusometry is monitoring changes in the resonance frequency of NMR active nuclei (such as ^1^H or ^19^F) associated with different values of the magnetic field experienced by the nuclei as a result of the diffusion of the ions. This method provides self-diffusion coefficients in contrast to NMR relaxometry that probes relative cation-cation, cation-anion, and anion-anion translation movement [10,11,12,13,14,15,16,17,18,19]. NMR relaxometry refers to magnetic field (and, hence, resonance frequency) dependent spin-lattice relaxation experiments, typically carried out in the frequency range from about 10 to 40 MHz (for ^1^H) [20,21]. In this context, one should point out two subjects. The first one is that NMR relaxation is caused (at least for nuclei of the spin quantum number 1/2, such as ^1^H or ^19^F) by mutual magnetic dipole–dipole interactions [22,23,24,25]. In case the interacting nuclei belong to different ions, the dipole–dipole coupling is modulated by the relative translation diffusion of the interacting species. Consequently, as already anticipated, NMR relaxometry gives access to the relative translation diffusion that, in the case of uncorrelated motion, is given as a sum of the self-diffusion coefficients of the participating ions. Thus, by comparing the results of NMR diffusometry and relaxometry, one can enquire into correlation effects in the translation diffusion [19]. The second subject concerns the time scale of the dynamics. At a given resonance frequency, the most efficient relaxation pathway is associated with a dynamical process occurring on a time scale matching the reciprocal resonance frequency [22,23,24,25]. This statement should not be treated as an “arbitrary truth” because in the case of several relaxation mechanisms (pathways), the relative amplitudes of the spin interactions are of importance. Nevertheless, it captures the essential fact that with increasing the magnetic field (and, hence, the resonance frequency), one probes moLecule (ionic) dynamics of progressively shorter time scales. Consequently, at low resonance frequencies, one gets access to translation diffusion, at intermediate frequencies, one probes rotational motion, while at high frequencies, one gets access to internal dynamics of molecules (ions). However, one should keep in mind that it is not possible to specify the low, intermediate, and high frequency ranges as amplitudes of the relevant spin interactions affect the contributions of the individual relaxation pathways to the overall relaxation process.

In this work we focus on internal dynamics of a series of ionic liquids: butyltriethylammonium bis(trifluoromethanesulfonyl)imide ([TEA-C4][TFSI])—C_12_H_24_F_6_N_2_O_2_S_2_, triethyloctylammonium bis(trifluoromethanesulfonyl)imide ([TEA-C8][TFSI])—C_16_H_32_F_6_N_2_O_2_S_2_, dodecyltriethylammonium bis(trifluoromethanesulfonyl)imide ([TEA-C12][TFSI])—C_20_H_40_F_6_N_2_O_2_S_2_ and hexadecyltriethylammonium bis(trifluoromethanesulfonyl)imide ([TEA-C16][TFSI])—C_24_H_48_F_6_N_2_O_2_S_2_. The liquids include the same anion, while the cations differ with respect to the length of the alkyl chain. It has already been shown that thermodynamical properties of these liquids are considerably affected by the alkyl chain length [26]. With the knowledge gained from the thermodynamic studies, here we focus on the temperature evolution of the internal dynamics of the cations and the anion over a very broad temperature range from about 100 K to about 400 K covering several phase transitions of the liquids. The internal dynamics of the cations and the anion are investigated by means of ^1^H and ^19^F (for the cations and the anion, respectively) NMR relaxation studies performed at 200 MHz (referring to the ^1^H resonance frequency). The large data set has been quantitatively analyzed assuming Arrhenius dependencies of the characteristic time constants (correlation times) in contrast to often used phenomenological models (such as Cole–Davidson or Havriliak–Negami spectral density functions [27,28,29]) that involve distributions of correlation times that, in our opinion, can hardly be justified. The fact that one can satisfactory reproduce the relaxation data not resourcing to models assuming heterogeneous dynamics is, beside the information about the internal dynamics of the ions, an important outcome of this work.

## 2. Theory

According to the spin relaxation theory, the spin-lattice relaxation rate, $R_{1}\left(\omega \right)$, caused by magnetic dipole–dipole interactions for a system of identical (equivalent) nuclei is given as [22,23,24,25]:

(1) $$R_{1}\left(\omega \right)=C_{DD}\left[J\left(\omega \right)+4J\left(2\omega \right)\right]$$

where $C_{DD}$ denotes the dipole–dipole relaxation constant, while J(ω) is referred to as a spectral density function being Fourier transform of the corresponding correlation function associated with the dynamical process that modulates the dipole–dipole interaction causing the relaxation. For exponential correlation function the spectral density is of the Lorentzian form and, consequently, Equation (1) can explicitly be written as [22,23,24,25]:

(2) $$R_{1}\left(\omega \right)=C_{DD}\left[\frac{\tau_{c}}{1+{\left(\omega \tau_{c}\right)}^{2}}+\frac{4\tau_{c}}{1+{\left(2\omega \tau_{c}\right)}^{2}}\right]$$

where $\tau_{c}$ denotes the correlation time characterizing the fluctuations of the dipole–dipole interaction. As anticipated in the Section 1, we assume that the correlation time follows the Arrhenius dependence:

(3) $$\tau_{c}=\tau_{0}exp\left(\frac{E_{A}}{RT}\right)$$

where $E_{A}$ denotes the activation energy, $\tau_{0}$ is the high temperature limit of the correlation time, while R denotes gas constant. In case there is more contributions to the overall relaxation rate (for instance two), Equation (2) can straightforwardly be modified to account for this effect, then:

(4) $$R_{1}\left(\omega \right)=C_{DD,1}\left[\frac{\tau_{c,1}}{1+{\left(\omega \tau_{c,1}\right)}^{2}}+\frac{4\tau_{c,1}}{1+{\left(2\omega \tau_{c,1}\right)}^{2}}\right]+C_{DD,2}\left[\frac{\tau_{c,2}}{1+{\left(\omega \tau_{c,2}\right)}^{2}}+\frac{4\tau_{c,2}}{1+{\left(2\omega \tau_{c,2}\right)}^{2}}\right]$$

where the pairs of the parameters: $C_{DD,1}$, $\tau_{c,1}$ and $C_{DD,2}$, $\tau_{c,2}$ characterize the dynamical processes associated with the corresponding relaxation contributions. In analogy to Equation (3), the correlation times evolve with temperature with the corresponding values of the activation energies and their high temperature limits.

## 3. Materials and Methods

The synthesis procedure as well as the thermodynamical characteristics of the ionic liquids: [TEA-C4][TFSI] (butyltriethylammonium bis(trifluoromethanesulfonyl)imide), [TEA-C8][TFSI] (triethyloctylammonium bis(trifluoromethanesulfonyl)imide), [TEA-C12][TFSI] (dodecyltriethylammonium bis(trifluoromethanesulfonyl)imide) and [TEA-C16][TFSI] (hexadecyltriethylammonium bis(trifluoromethanesulfonyl)imide) are described in [26]. Triethylamine, alkyl bromides (with 4, 8, 12, and 16 carbon atoms in the alkyl chain), lithium bis(trifluoromethanesulfonyl)imide, as well as all solvents were purchased from commercial suppliers (Merck KGaA, Darmstadt, Germany, Avantor Performance Materials Poland S.A., Gliwice, Poland, Acros Organics B.V.B.A., Delphi, India) and used without further purification.

^1^H and ^19^F spin-lattice relaxation experiments were performed using Bruker CXP (Billerica, MA, USA) spectrometer operating at the frequency of 200 MHz (for ^1^H). The spectrometer includes a cryogenic superconducting magnet produced by Oxford-Instruments, generating\inductance field of 4.7T. The relaxation experiments were carried out in the temperature range from 383 K (the samples were heated up to that temperature) down to 108 K with the step of 5 K (±1 K). The temperature was controlled using a gas-flow cryostat and monitored by Pt resistance thermometers with accuracy better than 1 K (determined with previously temperature-calibrated measuring systems). The samples were allowed to thermalize for 20 min before each experiment. The relaxation processes have turned out to be single exponential (saturation recovery sequence was used).

**Scheme 1:**
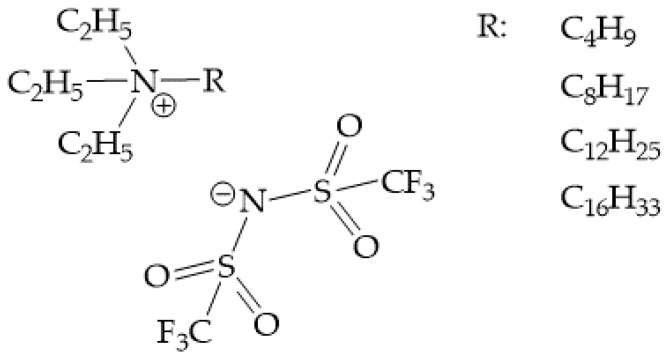
Ionic liquids used in this study.

The structural formula of the ionic liquids is presented below (Scheme 1).

## 4. Results

**Figure 1:**
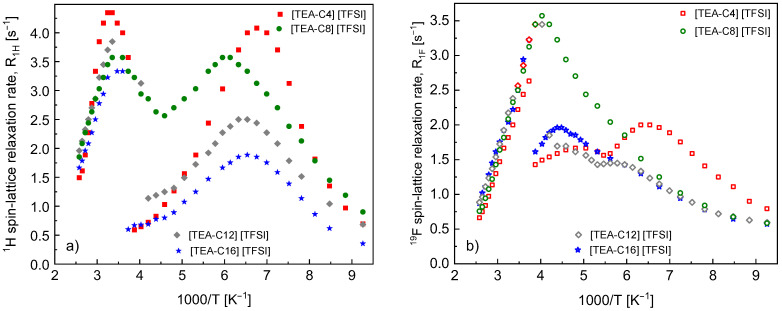
^1^H (**a**) and ^19^F (**b**) spin-lattice relaxation rates versus reciprocal temperature for [TEA-C4][TFSI], [TEA-C8][TFSI], [TEA-C12][TFSI] and [TEA-C16][TFSI].

^1^H and ^19^F spin-lattice relaxation rates collected in a very broad temperature range are shown in Figure 1.

The first observation is that the relaxation features show considerable diversity—the changes in the cations’ structure have a significant impact on both ^1^H and ^19^F relaxation properties. The second observation is discontinuity of the temperature dependencies of the relaxation rates observed for [TEA-C4][TFSI], [TEA-C12][TFSI] and [TEA-C16][TFSI], but not for [TEA-C8][TFSI].

**Figure 2:**
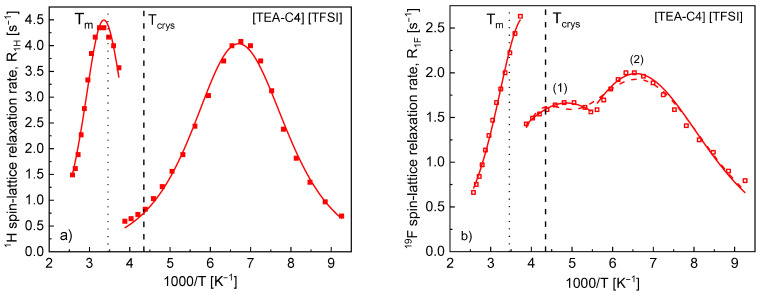
^1^H (**a**) and ^19^F (**b**) spin-lattice relaxation rates versus reciprocal temperature for [TEA-C4] [TFSI]. Solid lines—fits with parameters collected in Table 1, dashed red line—fit with parameters give in the text, vertical lines indicate phase transition temperatures ($T_{crys}$ = 229.89 K and $T_{m}$ = 289.10 K denote the crystallization and melting temperatures [26], respectively).

We begin the analysis of the relaxation data with ^1^H relaxation for [TEA-C4][TFSI]. It has turned out that one can reproduce the relaxation data at high as well as low temperatures (Figure 2a) in terms of a single dynamical process, i.e., in terms of Equation (2) (with the correlation time of Equation (3)).

The discontinuity of the relaxation rates is placed between the melting and the crystallization temperatures [26]. The obtained parameters are collected in Table 1; the indices “*h*” and “*l*” refer to the high and low temperature ranges, respectively—i.e., the ranges above and below the discontinuity temperature. The ^19^F spin-lattice relaxation data analysis has turned out to be more demanding, as expected from the shape of the temperature dependence of the relaxation rate. The data in the high temperature range can be well reproduced in terms of a single dynamical process (Figure 2b). However, below the discontinuity temperature, the data show two maxima that implies the presence of (at least) two motional processes associated with the relaxation. One can imagine two scenarios. In the first one, there are two kinds of motion, the correlation times of which follow the Arrhenius dependence in the whole temperature range (Equation (3)) above the discontinuity temperature. This approach does not lead to a satisfactory agreement with the experimental data (dashed red line in Figure 2b); the parameters of the fit are: ($C_{DD}=$ 1.69 × 10^9^ Hz^2^, $E_{A}=$ 5.81 kJ/moL, $\tau_{0}$ = 4.61 × 10^−12^ s) and ($C_{DD}=$ 7.84 × 10^8^ Hz^2^, $E_{A}=$ 0.14 kJ/moL, $\tau_{0}$ = 5.82 × 10^−13^ s). One can think about extending the model by replacing the Arrhenius dependence with the Vogel–Fulcher–Tammann equation [30], but this means including to the description two more parameters. Instead of that, at this stage, we have reproduced the data in terms of Equation (2) with different parameters for the ranges of 158–183 K (indicated in Figure 2b as (1)) and 178–108 K (indicated in Figure 2b as (2)). The obtained parameters are included in Table 1 with the corresponding indices.

**Figure 3:**
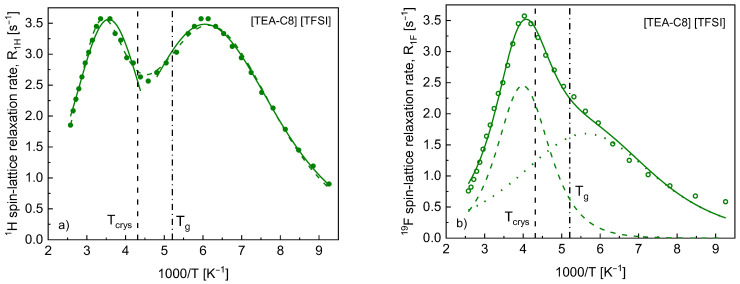
^1^H (**a**) and ^19^F (**b**) spin-lattice relaxation rates versus reciprocal temperature for [TEA-C8][TFSI]. Solid and dashed green lines—fits with parameters collected in Table 2, dashed line in (**b**)—relaxation contribution related to the parameters $C_{DD,1}^{}$, $\tau_{0,1}^{}$, $E_{A,1}^{}$, dotted line in (**b**)—relaxation contribution related to the parameters $C_{DD,2}^{}$, $\tau_{0,2}^{}$, $E_{A,2}^{}$, vertical lines indicate phase transition temperatures ($T_{crys}\;$ = 231.83 K, the glass transition temperature $T_{g}\;$ = 192.00 K [26]).

Following this line, Figure 3a,b shows the result of the analysis of the ^1^H and ^19^F spin-lattice relaxation data for [TEA-C8][TFSI]. As already pointed out, [TEA-C8][TFSI] is the only case for which one does not observe a discontinuity in the temperature dependencies of the relaxation rates.

Independently of that, for the ^1^H relaxation, one can still use the terminology described in the context of the analysis of the relaxation data for [TEA-C4][TFSI], referring to the high and low temperatures ranges that correspond to the relaxation maxima seen in Figure 3a. In the first step, we have separately reproduced the relaxation data in the temperature ranges of 329–228 K and 219–108 K in terms of Equation (1). This concept is supported by the crystallization temperature of [TEA-C8][TFSI], 231.83K, being close to the limit of the high temperature range (228 K). The obtained parameters are collected in Table 2. In the next step, motivated by the continuous dependence of the ^1^H spin-lattice relaxation rate on temperature, we have attempted to reproduce the relaxation data in the whole temperature range in terms of Equation (3) (dashed green line in Figure 3a). The same approach (Equation (3)) has been applied to reproduce the temperature dependence of ^19^F spin-lattice relaxation rates (Figure 3b). The parameters are collected in Table 2; the dashed line in Figure 3b corresponds to the set of parameters $C_{DD,1}^{}$, $\tau_{0,1}^{}$, $E_{A,1}^{}$.

**Figure 4:**
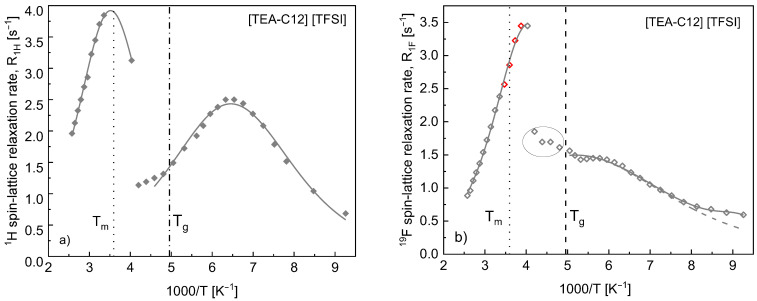
^1^H (**a**) and ^19^F (**b**) spin-lattice relaxation rates versus reciprocal temperature for [TEA-C12][TFSI]. Solid lines—fits with parameters collected in Table 3, dashed line in (**b**)—relaxation contribution related to the parameters $C_{DD,2}^{l}$, $\tau_{0,2}^{l}$, $E_{A,2}^{l}$, vertical lines indicate phase transition temperatures ($T_{m}\;$ = 278.20 K, $T_{g}\;$ = 201.77 K [26]).

The ^1^H spin-lattice relaxation data for [TEA-C12][TFSI] can satisfactorily be interpreted in terms of Equation (1) applied to the high and low temperature ranges (Figure 4a).

The temperature dependence of the ^19^F spin-lattice relaxation also shows a discontinuity and high temperature part follows Equation (2). The shape of the low temperature part indicates the presence of more dynamical processes. The outcome of applying Equation (3) is shown in Figure 4b, while all obtained parameters are collected in Table 3. We have omitted in the analysis of the ^19^F spin-lattice relaxation data the four points circled in Figure 3b. To reproduce this part of the temperature dependence of the ^19^F spin-lattice relaxation rates one would need an additional set of parameters with high uncertainty due to the small number of the experimental points. One can also see from Figure 3b that the relaxation contribution corresponding to the parameters $C_{DD,2}^{l}$, $\tau_{0,2}^{l}$, $E_{A,2}^{l}$ becomes relevant only at very low temperatures.

**Figure 5:**
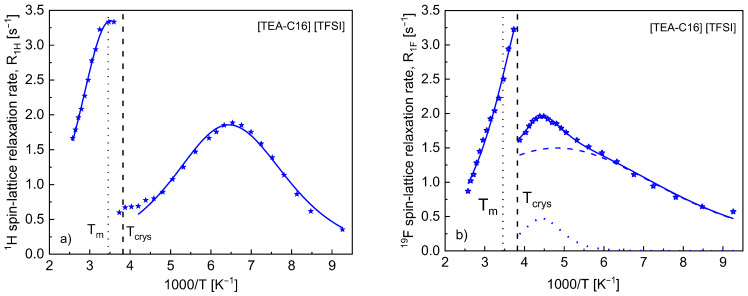
^1^H (**a**) and ^19^F (**b**) spin-lattice relaxation rates versus reciprocal temperature for [TEA-C16][TFSI]. Solid and dashed green lines—fits with parameters collected in Table 4, dashed line in (**b**)—relaxation contribution related to the parameters $C_{DD,1}^{l}$, $\tau_{0,1}^{l}$, $E_{A,1}^{l}$, dotted line in (**b**)—relaxation contribution related to the parameters $C_{DD,2}^{l}$, $\tau_{0,2}^{l}$, $E_{A,2}^{l}$,vertical lines indicate phase transition temperatures ($T_{m}\;$ = 289.41 K, $T_{cryst}\;$ = 261.69 K [26]).

The relaxation data for [TEA-C16][TFSI] have been interpreted in the same manner as those for [TEA-C12][TFSI]. The results of the fits are shown in Figure 5a,b for ^1^H and ^19^F, respectively, while the obtained parameters are collected in Table 4.

**Figure 6:**
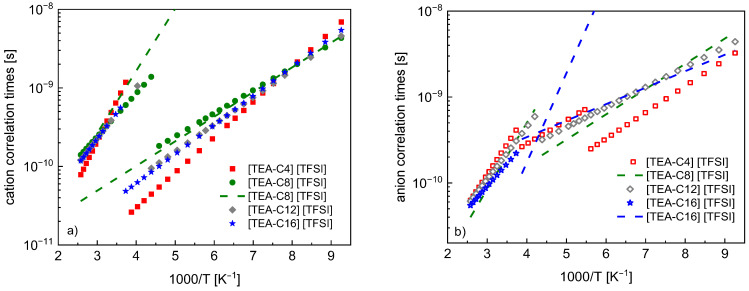
Correlation times for the cations (**a**) and the anion (**b**) versus reciprocal temperature; for (**a**): red squares—correlation times corresponding to the fit of Figure 2a, green circles—correlation times corresponding to the fit represented in Figure 3a as solid lines, dashed green lines—correlation times corresponding to the fit represented in Figure 3a as dashed line, grey diamonds—correlation times corresponding to the fit of Figure 4a, blue stars—correlation times corresponding to the fit of Figure 4a; for (**b**): red squares—correlation times corresponding to the fit represented in Figure 2b as solid lines, green dashed lines—correlation times corresponding to the fit of Figure 3b, grey diamonds—correlation times corresponding to the fit of Figure 4a, blue stars—correlation time corresponding to the fit of Figure 4b below $T_{crys}$, dashed blue lines—correlation times corresponding to the fit of Figure 4b above $T_{crys}$.

On the basis of the obtained parameters the temperature dependencies of the correlation times have been simulated in Figure 6a,b for the cations and the anion, respectively.

The results are discussed in the next section.

## 5. Discussion

As already pointed out, the ^1^H spin-lattice relaxation data for [TEA-C4][TFSI] show a discontinuity between the crystallization and melting temperatures. The two parts of the temperature dependence of the relaxation rate have been interpreted in terms of dynamical processes following the Arrhenius law. The simulated temperature dependencies of the correlation times of [TEA-C4] cations at temperatures above and below the discontinuity are shown in Figure 6a (red points). Comparing the correlation time in the high temperature range with the rotational correlation time of [TEA-C4] cations in [TEA-C4][TFSI] [19] one sees that the last one is significantly longer—consequently, at 200 MHz one indeed probes the internal dynamics of the cation, not its rotation. Thus, one can suppose that the correlation time characterizes the motion of C_2_H_5_ chains in the [TEA-C4] cation. Upon the phase transition the dynamics of the chains slows down, and the relaxation becomes governed by the methyl group rotation at temperatures below the discontinuity. In principle, one can imagine a similar scenario for all the liquids—however, with some variations. For [TEA-C8][TFSI] one does not observe a discontinuity in the relaxation curve. This effect is, however, misguiding. The analysis (solid lines in Figure 3b) shows that to reproduce the data two dynamical processes are required (that is obviously taking into account the presence of the two maxima of the relaxation rate) and the corresponding correlation times do not match each other (green points in Figure 6a)—there is a gap between them, in analogy to the case of [TEA-C4][TFSI]. The attempt to reproduce the data in a continuous manner (dashed line in Figure 3b) has led to a similar output in this sense that the two correlation times (dashed green lines in Figure 6b) do not differ much from those obtained from reproducing the relaxation maxima separately. It is worth noting that for [TEA-C12][TFSI] one sees a discontinuity in the relaxation rate, although for this liquid a crystallization temperature has not been determined [26]. Looking at the high temperature wing of the correlation times one can observe that the correlation times for [TEA-C8][TFSI], [TEA-C12][TFSI] and [TEA-C16][TFSI] almost overlap—some differences are observed for [TEA-C4][TFSI]. In the [TEA-C8], [TEA-C12] and [TEA-C16] cations one of the alkyl chains becomes progressively longer compared to the three C_2_H_5_ chains, while [TEA-C4] is similar in the chain length. In the low temperature range the correlation times for [TEA-C12] and [TEA-C16] almost overlap, while some differences are observed for [TEA-C4] and [TEA-C8]. Although we attribute the correlation times in this range mainly to the methyl group rotation, one can expect that the chain dynamics somewhat mediates the results. With decreasing temperature, when the chain dynamics become progressively slower, the correlation times for all cations converge. It is also of interest to note that the dipolar relaxation constants, $C_{DD}^{h}$ and $C_{DD}^{l}$ for [TEA-C4] and [TEA-C8] are similar (this is reflected by the similar values of the high temperature and low temperature relaxation maxima (Figure 3a and Figure 4a), while for [TEA-C12] and [TEA-C16] the dipolar relaxation constant $C_{DD}^{l}$ becomes progressively lower compared to $C_{DD}^{h}$. This can be explained by the presence of the ^1^H nuclei in the progressively longer alkyl chain that act as a “magnetization sink” because of their relatively slow motion [31].

Discussing the dynamics of the [TFSI] anion, one should again begin with the observation that the high temperature wing of the correlation times (Figure 6b) is not associated with rotational dynamics of the anion—the rotation is slower [19]. Thus, one can expect, starting with the case of [TEA-C4][TFSI], that the high temperature wing of the correlation times correspond to internal dynamics of the anion. Then, in analogy to the cation dynamics, at lower temperatures (below the discontinuity of the temperature dependence of the ^19^F spin-lattice relaxation rate) one probes the correlation time of the CF_3_ group rotation. However, in this temperature range one clearly sees two maxima of the relaxation rates, and this implies the presence of two dynamical processes, independently of the details of the analysis. The simulated, corresponding correlation times are shown in Figure 6b (red points). A possible explanation of this finding is the presence of two fractions of [TFSI] anions undergoing somewhat different dynamics. The ^19^F spin-lattice relaxation data for [TEA-C8][TFSI] does not show any discontinuity and they can be reproduced in terms of two dynamical processes present over the whole temperature range—the corresponding correlation times are shown in Figure 6b as green lines (lines were used to underline the “continuous” character of the dynamics). One can see that one branch of the correlation times matches the part attributed to the internal dynamics of the [TFSI] anion (not related to the CF_3_ group rotation), while the second branch converges at low temperatures to the correlation time of the CF_3_ group rotation. One could say that for [TEA-C8][TFSI] the internal dynamics of the [TFSI] cation remains unaffected by crystallization. The correlation times for [TFSI] anions in [TEA-C12][TFSI] follow the scenario—the internal (not CF_3_ rotation) dynamics at high temperatures and CF_3_ group rotation at high temperatures. It is of interest to note that to reproduce the data at very low temperatures a dynamical process of a very short correlation time is required. The corresponding relaxation contribution is neither explicitly shown in Figure 4b, nor the corresponding correlation time is shown in Figure 6b (in fact, a similar effect is observed for [TEA-C8][TFSI] (Figure 3b)). Eventually, the correlation time for [TFSI] in [TEA-C16][TFSI] in the high temperature range follows the pattern attributed to the internal dynamics of the anion (solid points in Figure 6b), however, below the crystallization temperature two intertwined dynamical processes are present (blue lines in Figure 6b)—one of them being the CF_3_ group rotation, while the second one likely represent the internal dynamics, in this case affected by the phase transition.

## 6. Conclusions

^1^H and ^19^F spin-lattice relaxation experiments have been performed for a series of ionic liquids composed of [TFSI] anion and [TEA-C4], [TEA-C8], [TEA-C12] and [TEA-C16] cations at the resonance frequency of 200 MHz (referring to ^1^H) in a broad temperature range from about 100 K to about 400 K. The data have been interpreted in terms of a relaxation model assuming the Arrhenius law for the correlation times characterizing the dynamical processes involved in the relaxation. As far as the cation dynamics is concerned, two dynamical processes have been revealed—the dynamics of the alkyl chains and the CH_3_ group rotation; the second process becomes efficient as the relaxation mechanism at lower temperatures. This scenario applies to all the liquids, although the ^1^H spin-lattice relaxation data for [TEA-C8] do not show any discontinuity at the phase transition. The correlation times associated with the chain dynamics in [TEA-C8], [TEA-C12] and [TEA-C16] are very close; some differences are observed for [TEA-C4]. The correlation times attributed to the methyl group rotation converge at low temperatures when they are not affected anymore by the chain dynamics. It has turned out that the structure of the cations considerably affects the dynamics of the [TFSI] anion. On the basis of the ^19^F spin-lattice relaxation data, two dynamical processes of the anion have been revealed: an internal motion of the anion (we would prefer not to speculate with respect to the specific geometry of the motion) and the CF_3_ group rotation that manifests itself mostly at low temperatures. The results indicate that for [TEA-C4][TFSI] there are two fractions of TFSI anions with different internal mobility (in terms of the correlation times of this motion). In the case of [TEA-C8][TFSI] and [TEA-C16][TFSI] the two dynamical processes are present in the whole temperature range in this sense that the internal dynamics does not considerably slow down upon the phase transition (in fact, it seems that for [TEA-C8][TFSI] the process remains unaffected by crystallization).

The two main conclusions from the detailed analysis of the relaxation data are: the internal dynamics of the ions follows the Arrhenius law in a very broad temperature range, and changes in the structure of the cation affect the dynamics of both species: the cation and the anion.

## Figures and Tables

**Table 1:** Parameters obtained from the analysis of the ^1^H and ^19^F spin-lattice relaxation data for [TEA-C4][TFSI].

[TEA-C4][TFSI]						
^1^H	$C_{DD}^{h}$ **[Hz^2^]**	$\tau_{0}^{h}$ **[s]**	$E_{A}^{h}$ **(kJ/moL)**	$C_{DD}^{l}$ **(Hz^2^)**	$\tau_{0}^{l}$ **(s)**	$E_{A}^{l}$ **(kJ/moL)**
	3.96 × 10^9^ (±7.7 × 10^7^)	1.87 × 10^−13^ (±5.8 × 10^−14^)	19.49 (±0.82)	3.56 × 10^9^ (±4.0 × 10^7^)	4.73 × 10^−13^ (±5.2 × 10^−14^)	8.61 (±0.14)
^19^F	$C_{DD}^{h}$ **[Hz^2^]**	$\tau_{0}^{h}$ **[s]**	$E_{A}^{h}$ **(kJ/moL)**	$C_{DD}^{l\left(1\right)}$ **(Hz^2^)**	$\tau_{0}^{l\left(1\right)}$ **(s)**	$E_{A}^{l\left(1\right)}$ **(kJ/moL)**
				$C_{DD}^{l\left(2\right)}$ **(Hz^2^)**	$\tau_{0}^{l\left(2\right)}$ **(s)**	$E_{A}^{l\left(2\right)}$ **(kJ/moL)**
	2.32 × 10^9^ (±4.9 × 10^7^)	9.36 × 10^−13^ (±1.6 × 10^−13^)	13.54 (±0.51)	1.46 × 10^9^ (±5.4 × 10^6^)	2.34 × 10^−11^ (±2.2 × 10^−12^)	5.20 (±0.17)
				1.75 × 10^9^ (±2.9 × 10^7^)	4.76 × 10^−12^ (±7.6 × 10^−13^)	5.86 (±0.18)

**Table 2:** Parameters obtained from the analysis of the ^1^H and ^19^F spin-lattice relaxation data for [TEA-C8][TFSI].

[TEA-C8][TFSI]						
^1^H	$C_{DD}^{h}$ **[Hz^2^]**	$\tau_{0}^{h}$ **(s)**	$E_{A}^{h}$ **(kJ/moL)**	$C_{DD}^{l}$ **(Hz^2^)**	$\tau_{0}^{l}$ **(s)**	$E_{A}^{l}$ **(kJ/moL)**
	3.14 × 10^9^ (±4.5 × 10^7^)	5.38 × 10^−12^ (±8.6 × 10^−13^)	10.52 (±0.39)	3.07 × 10^9^ (±1.9 × 10^7^)	8.16 × 10^−12^ (±6.1 × 10^−13^)	5.63 (±0.09)
	$C_{DD,1}^{}$ **[Hz^2^]**	$\tau_{0,1}^{}$ **(s)**	$E_{A,1}^{}$ **(kJ/moL)**	$C_{DD,2}^{}$ **(Hz^2^)**	$\tau_{0,2}^{}$ **(s)**	$E_{A,2}^{}$ **(kJ/moL)**
	2.30 × 10^9^ (±4.1 × 10^7^)	1.12 × 10^−12^ (±2.1 × 10^−13^)	15.16 (±0.48)	3.03 × 10^9^ (±2.2 × 10^7^)	5.62 × 10^−12^ (±5.8 × 10^−13^)	6.01 (±0.12)
^19^F	$C_{DD,1}^{}$ **[Hz^2^]**	$\tau_{0,1}^{}$ **(s)**	$E_{A,1}^{}$ **(kJ/moL)**	$C_{DD,2}^{}$ **(Hz^2^)**	$\tau_{0,2}^{}$ **(s)**	$E_{A,2}^{}$ **(kJ/moL)**
	2.16 × 10^9^ (±2.7 × 10^8^)	4.10 × 10^−13^ (±3.7 × 10^−14^)	14.77 (±1.91)	1.48 × 10^9^ (±1.6 × 10^8^)	1.08 × 10^−11^ (±6.0 × 10^−12^)	5.63 (±0.52)

**Table 3:** Parameters obtained from the analysis of the ^1^H and ^19^F spin-lattice relaxation data for [TEA-C12][TFSI].

[TEA-C12][TFSI]						
^1^H	$C_{DD}^{h}$ **(Hz^2^)**	$\tau_{0}^{h}$ **(s)**	$E_{A}^{h}$ **(kJ/moL)**	$C_{DD}^{l}$ **(Hz^2^)**	$\tau_{0}^{l}$ **(s)**	$E_{A}^{l}$ **(kJ/moL)**
	3.45 × 10^9^ (±3.1 × 10^7^)	2.56 × 10^−12^ (±2.3 × 10^−13^)	12.42 (±0.22)	2.14 × 10^9^ (±2.8 × 10^7^)	2.88 × 10^−12^ (±4.0 × 10^−13^)	6.62 (±0.18)
^19^F	$C_{DD}^{h}$ **(Hz^2^)**	$\tau_{0}^{h}$ **[s]**	$E_{A}^{h}$ **(kJ/moL)**	$C_{DD,1}^{l}$ **(Hz^2^)**	$\tau_{0,1}^{l}$ **(s)**	$E_{A,1}^{l}$ **(kJ/moL)**
				$C_{DD,2}^{l}$ **(Hz^2^)**	$\tau_{0,2}^{l}$ **(s)**	$E_{A,2}^{l}$ **(kJ/moL)**
	3.07 × 10^9^ (±3.8 × 10^7^)	1.68 × 10^−12^ (±1.8 × 10^−13^)	11.61 (±0.30)	1.90 × 10^8^ (±4.4 × 10^7^)	1.17 × 10^−17^ (±4.1 × 10^−18^)	15.82 (±2.40)
				1.32 × 10^9^ (±1.8 × 10^7^)	3.0 × 10^−11^ (±1.4 × 10^−11^)	4.48 (±0.63)

**Table 4:** Parameters obtained from the analysis of the ^1^H and ^19^F spin-lattice relaxation data for [TEA-C16][TFSI].

[TEA-C16][TFSI]						
^1^H	$C_{DD}^{h}$ **[Hz^2^]**	$\tau_{0}^{h}$ **(s)**	$E_{A}^{h}$ **(kJ/moL)**	$C_{DD}^{l}$ **(Hz^2^)**	$\tau_{0}^{l}$ **(s)**	$E_{A}^{l}$ **(kJ/moL)**
	2.95 × 10^9^ (±2.3 × 10^7^)	2.35 × 10^−12^ (±3.6 × 10^−13^)	12.64 (±0.44)	1.63 × 10^9^ (±2.3 × 10^7^)	1.99 × 10^−12^ (±2.6 × 10^−13^)	7.10 (±0.17)
^19^F	$C_{DD}^{h}$ **[Hz^2^]**	$\tau_{0}^{h}$ **(s)**	$E_{A}^{h}$ **(kJ/moL)**	$C_{DD,1}^{l}$ **(Hz^2^)**	$\tau_{0,1}^{l}$ **(s)**	$E_{A,1}^{l}$ **(kJ/moL)**
				$C_{DD,2}^{l}$ **(Hz^2^)**	$\tau_{0,2}^{l}$ **(s)**	$E_{A,2}^{l}$ **(kJ/moL)**
	3.64 × 10^9^ (±6.2 × 10^8^)	2.42 × 10^−12^ (±3.9 × 10^−13^)	10.06 (±0.84)	1.32 × 10^9^ (±1.4 × 10^8^)	5.84 × 10^−11^ (±1.9 × 10^−11^)	3.67 (±0.25)
				4.15 × 10^8^ (±1.3 × 10^8^)	1.22 × 10^−14^ (±5.9 × 10^−11^)	19.87 (±8.69)

## Data Availability

All data are available from the corresponding author.
